# Is there still a role for ultrasound in trauma?

**DOI:** 10.1186/1757-7241-23-S2-A19

**Published:** 2015-09-11

**Authors:** Jamie Moran, Tim Harris, Jeremy Purdell-Lewis

**Affiliations:** 1 Emergency Department, Royal London Hospital Barts Health NHS Trust, London, UK

## Background

The UK College of Emergency Medicine recommends that level 1 ultrasound competency is a basic standard for EM doctors and is now mandatory for career progression. Focused Assessment with Sonography in Trauma to include the detection of pleural fluid and pneumothorax (the Extended-FAST scan) forms part of this competency. We compare the diagnostic accuracy of E-FAST with the “gold standard” of CT or operative intervention. Trauma team leaders were asked to evaluate point-of-care ultrasound in their decision-making and patient management.

## Methods

### Setting

Royal London Hospital. The Major Trauma Centre for North East London and base for London's Air Ambulance. Approximately 2500 adult trauma cases seen per year.

### Study design

Prospective observational study, comprising convenience sample of adult major trauma presenting to Royal London between October 2012-March 2013 leading to activation of a “trauma call”.

### Primary outcome

To assess the diagnostic accuracy of E-FAST in the detection of haemorrhage (free fluid) and pneumothorax in major trauma.

### Secondary outcome

To assess the impact of E-FAST on trauma team leader's decision-making process in major trauma care.

### Reference Standard

Free fluid or pneumothorax formally reported on CT or found at time of surgical intervention

## Results

117 patients initially recruited, 45 allowed comparison to reference standard. Sensitivity, Specificity, Positive and Negative Predictive Values for E-FAST (with 95% confidence intervals) were 68.4% (43.5-87.4), 96.3% (81-99.4), 92.9% (66.1-98.8) and 81.3% (63.6-92.8) respectively. 58% of team leaders stated that ultrasound guided their decision-making.

## Conclusion

E-FAST has limited sensitivity but high specificity when used in isolation. It influenced trauma team leader's decision-making 58% of the time, despite reported low sensitivity. The major role of ultrasound is the rapid triage of unstable patients and localization of major haemorrhage to help guide immediate life-saving intervention in this subgroup of patients. May reduce CT load in selected patients but further research needed.

